# Nephropathogenic Infectious Bronchitis Virus Infection Altered the Metabolome Profile and Immune Function of the Bursa of Fabricius in Chicken

**DOI:** 10.3389/fvets.2020.628270

**Published:** 2021-01-21

**Authors:** Jun Kuang, Puzhi Xu, Yan Shi, Yitian Yang, Ping Liu, Shupeng Chen, Changming Zhou, Guyue Li, Yu Zhuang, Ruiming Hu, Guoliang Hu, Xiaoquan Guo

**Affiliations:** ^1^Jiangxi Provincial Key Laboratory for Animal Health, College of Animal Science and Technology, Jiangxi Agricultural University, Nanchang, China; ^2^School of Computer and Information Engineering, Jiangxi Agricultural University, Nanchang, China

**Keywords:** metabolome, inflammatory cytokines, bursa of fabricius, nephropathogenic infectious bronchitis virus, correlation analysis

## Abstract

Infectious bronchitis is a highly contagious, acute viral respiratory disease of chickens, regardless of the strain, and its infection may lead to considerable economic losses to the poultry industry. New nephropathogenic infectious bronchitis virus (NIBV) strains have increasingly emerged in recent years; hence, evaluating their infection-influenced immune function changes and the alteration of metabolite profiling is important. Initially, chickens were randomly distributed into two groups: the control group (Con) and the disease group (Dis). Here, the partial cytokines were examined, and the metabolome alterations of the bursa of Fabricius (BF) in NIBV infections in chickens were profiled by gas chromatography time-of-flight/mass spectrometry (GC-TOF/MS). The results revealed that the NIBV infection promotes the mRNA expression of inflammatory cytokines. Metabolic profile analysis indicated that clustering differed between the two groups and there were 75 significantly different metabolites detected between the two groups, suggesting that the host metabolism was significantly changed by NIBV infection. Notably, the following 12 metabolites were identified as the potential biomarkers: 3-phenyllactic acid, 2-deoxytetronic acid, aminomalonic acid, malonamide 5, uric acid, arachidonic acid, 2-methylglutaric acid, linoleic acid, ethanolamine, stearic acid, N-alpha-acetyl-l-ornithine, and O-acetylserine. Furthermore, the results of the correlation analysis showed that a strong correlation existed between metabolic biomarkers and inflammatory cytokines. Our results describe an immune and metabolic profile for the BF of chickens when infected with NIBV and provide new biomarkers of NIBV infection as potential targets and indicators of indicating therapeutic efficacy.

## Introduction

Infectious bronchitis virus (IBV) is a gamma coronavirus in the family Coronaviridae that consists of a positive-stranded RNA of 27.6 kb in length, which has been recognized as a pathogen of infectious bronchitis (IB), as well as respiratory and urogenital diseases in the commercial poultry industry worldwide (1–3). All strains of IBV can replicate on most chicken epithelial surfaces, such as those of the trachea, lungs, kidney, oviduct, alimentary, and proventriculus (4, 5). In the 1960s, the first case of nephropathogenic IBV (NIBV) strains was reported in the United States. In the past 20 years, NIBV strains have been the most prevalent IBV strains worldwide (6–8). Infected chickens showed decreased egg production and egg quality with accompanying secondary complications. Necropsy of chickens that died after NIBV infection revealed enlarged and pale kidneys, with urate deposits in the collecting tubule, thus making it a major cause of economic losses within the commercial poultry industry (9).

The bursa of Fabricius (BF) is a unique central humoral immune organ to birds and plays a significant role in B lymphocyte differentiation and maturation (10, 11). B lymphocytes will migrate and colonize in the peripheral lymphatic organs after fully differentiated in the BF to perform important immune functions. B lymphocytes become the only cells in the body that produce antibodies by converting into plasma cells, which play a key role in the immune response process (12). According to research reports, the BF plays the role of a primary lymphatic organ before the chicken is 5–6 weeks old and then as a peripheral lymphatic organ (13). This organ is mainly composed of 98% B lymphocytes, but infiltration of other cells (such as T cells and macrophages) has been observed in response to viral infection (14). Previous studies have found that the BF is one of the target organs for virus replication, and the virus titer reaches its peak between 7 and 10 days after IBV infection (15, 16). As we all know, cytokines are important mediators and regulators for the host to resist foreign antigens, and their function is mainly to coordinate the functional activity of immune system cells (17–19). Previous studies have confirmed that the BF is involved in the host response to infectious bursal disease virus (IBDV) infection (20–22), including virus infection caused cytokine expression changes. However, the cytokine changes in the chicken's BF during NIBV infection are still poorly understood.

It is well-known that the host immune response plays a key role in the initiation and outcome of infection (23). Metabolic pathways are also commonly involved in and direct the immune response and may have a wide range of effects in the pathogenesis of viruses (24, 25). An upcoming hot topic in immune system research is immune metabolism, which studies the influence of cell metabolism on immune cell function. Metabolomics, a quickly emerging “omics,” is the global analysis of the low molecular weight metabolites (typically <1,000 Da) in a biological sample (26). Since the middle of the last century, the central principle of biological information has been the translation from genes to transcripts to proteins (27). However, until recently, high-throughput systems biology has been used to study the downstream products of protein activities, namely metabolites. The metabolome is the overall combination of all endogenous and exogenous small molecules in a biological state that is a transient “snapshot” of cell metabolism and physiological activities (28). With the development of technology, the application range of metabolomic analysis has become more and more extensive, such as for disease diagnosis, the discovery of biomarkers, and the study of the metabolic pathways and their alterations caused by external factors (29). What is more valuable is that the analysis of changes in the metabolites has the effect of providing new biomarkers, thus making it possible to intervene earlier and to understand the mechanism of the disease.

Metabolic changes are the end-result of adaptive and defensive biochemical reactions that occur during infection. Therefore, to explore the possible host–pathogen relationships between the chicken's BF and NIBV infection, the infection model by infecting chickens with NIBV was first built. Then, viral replication and cytokine expression in the BFs were detected to evaluate the immune level. The BFs from the models were analyzed *via* metabolome profiling by gas chromatography time-of-flight/mass spectrometry (GC-TOF/MS) technology to deeply explore the metabolites involved in the NIBV infection response. Metabolome studies on BF, which reflect the dynamic changes in the biological process, were done and correlated with the cytokine expression level to help us elucidate the effects of NIBV on immune and metabolism. In addition, this study also aims to obtain the potential metabolic biomarkers that can be used to effectively diagnose viral infections.

## Materials and Methods

### Experimental Design

We randomly divided 240 healthy Hy-Line Variety Brown chickens into two experimental animal breeding rooms, control group (Con) and NIBV infection disease group (Dis). The birds in each breeding room were then randomly divided into three parallel groups. At 28 days old, each chicken in the Dis group was injected intranasally with 0.2 ml of 10^5^ median embryo lethal doses of strain SX9 (30), whereas in the Con group, 0.2 ml of sterile physiological saline was intranasally received at the same time. On the 10th day after infection, two chickens randomly chosen from each parallel group were euthanized by carbon inhalation. In a sterile environment, we quickly separated and collected the BF samples. The BF samples were gathered for reverse transcriptase-quantitative PCR (RT-qPCR) and GC-TOF/MS detection. All animal experiments were approved by the Institutional Animal Care and Use Committee of Jiangxi Agricultural University (Approval ID: JXAULL-2017003).

### Detection of Cytokine Expression by RT-qPCR

Total RNA was purified from the BF samples using RNAiso Plus (Takara, Japan). Then, NanoDrop 1,000 Spectrophotometer was used to detect the concentration and purity of RNA at a wavelength of 260–280 nm. cDNA was carried out with One-Step gDNA Removal and cDNA Synthesis SuperMix Kit (TransGen Biotech, China). The cDNA was stored at −20°C for real-time PCR. The primer sequences for the amplification of cytokine genes are shown in Table 1.

**Table 1:** Nucleotide sequences of specific primers.

**Gene**	**Accession no**.	**Products**	**Primer sequence**
IL-2	NM_204153	111 bp	F: 5′-GAACCTCAAGAGTCTTACGGGTCTA-3′
			R: 5′-ACAAAGTTGGTCAGTTCATGGAGA-3′
IL-6	NM_204628	106 bp	F: 5′-AAATCCCTCCTCGCCAATCT-3′
			R: 5′-CCCTCACGGTCTTCTCCATAAA-3′
IL-8	NM_205018	134 bp	F: 5′-GTGACACCCGGAAGAAACAC-3′
			R: 5′-CTGGCATCGGAGTTCAATCG-3′
IL-12β	NM_213571	160 bp	F: 5′-GAGCACACCGAAGTCCTACT-3′
			R: 5′-GTAATAGCGATCCCTGGCCT-3′
IL-18	NM_204608	94 bp	F: 5′-AGGTGAAATCTGGCAGTGGAAT-3′
			R: 5′-ACCTGGACGCTGAATGCAA-3′
TNF-α	NM_204267.1	268 bp	F: 5′-CAGATGGGAAGGGAATGAAC-3′
			R: 5′-AGAGCATCAACGCAAAAGGG-3′
IFN-α	NM_205427.1	75 bp	F: 5′-GGACATGGCTCCCACACTAC-3′
			R: 5′-TCCAGGATGGTGTCGTTGAAG-3′
IFN-γ	NM_205149	71 bp	F: 5′-GTGAAGAAGGTGAAAGATATCATGGA-3′
			R: 5′-GCTTTGCGCTGGATTCTCA-3′
CXCLi2	NM_205498	74 bp	F: 5′-GCCCTCCTCCTGGTTTCAG-3′
			R: 5′-TGGCACCGCAGCTCATT-3′
GAPDH	NM_204305	105 bp	F: 5′-GAACGGGAAACTTGTGAT-3′
			R: 5′-GACTCCACAACATACTCA-3′

PCR was carried out using QuantStudio 7 Flex Real-Time PCR system (ABI Thermo Fisher Scientific, USA) according to the instruction of TransStart Tip Green qPCR SuperMix (TransGen Biotech, China). The PCR system included 5 μl of Tip Green qPCR SuperMix (2×), 0.2 μl Forward and Reverse primers, 0.2 μl of Passive Referencing dye (50×), 1 μl of cDNA templates, and 3.4 μl of water. The amplification was denaturation step at 94°C for 30 s, followed by 40 cycles at 94°C for 5 s, 60°C for 30 s, and extension at 72°C for 30 s. Finally, the method of 2^−ΔΔ*Ct*^ was used to analyze the data and graphically with Prism software.

### Metabolome Analysis of the Chicken's BF With NIBV Infection

The detailed process of GC-TOF/MS analysis follows the method of Yang et al. (31). In short: (i) metabolite extraction was performed on six samples in each group, and l-2-chlorophenylalanine was added as an internal standard; (ii) metabolite derivatization uses the methoxyamine hydrochloride and the BSTFA reagent; (iii) the Agilent 7,890 gas chromatograph system coupled with a Pegasus HT time-of-flight mass spectrometer was used to detect metabolites. The mass spectrometry data were acquired with an m/z range of 50–500 at a rate of 20 spectra per second after a solvent delay of 6.04 min (−70 eV, full-scan mode).

Chroma TOF4.3X software of the LECO Corporation and the LECO-Fiehn Rtx5 database were used for data preprocessing. Then, the SIMCA14 software package (Umetrics, Umea, Sweden) was used to perform principal component analysis (PCA) and orthogonal projections to latent structures-discriminant analysis (OPLS-DA).

### Correlation Analysis of Inflammatory Cytokines and Metabolite Biomarkers

The correlation coefficient of inflammatory cytokines and metabolite biomarkers is carried out through the “Corrplot” package (https://cran.rproject.org/web/packages/corrplot/index.html) in R software. The method of correlation analysis is Spearman correlation. The value of the correlation coefficient is between −1 and +1. The magnitude of the value indicates the strength of the correlation relationship, “+” indicates a positive correlation between the two, and “–” indicates a negative correlation.

## Results

### NIBV Infection Promotes the mRNA Expression of Inflammatory Cytokines

**Figure 1:**
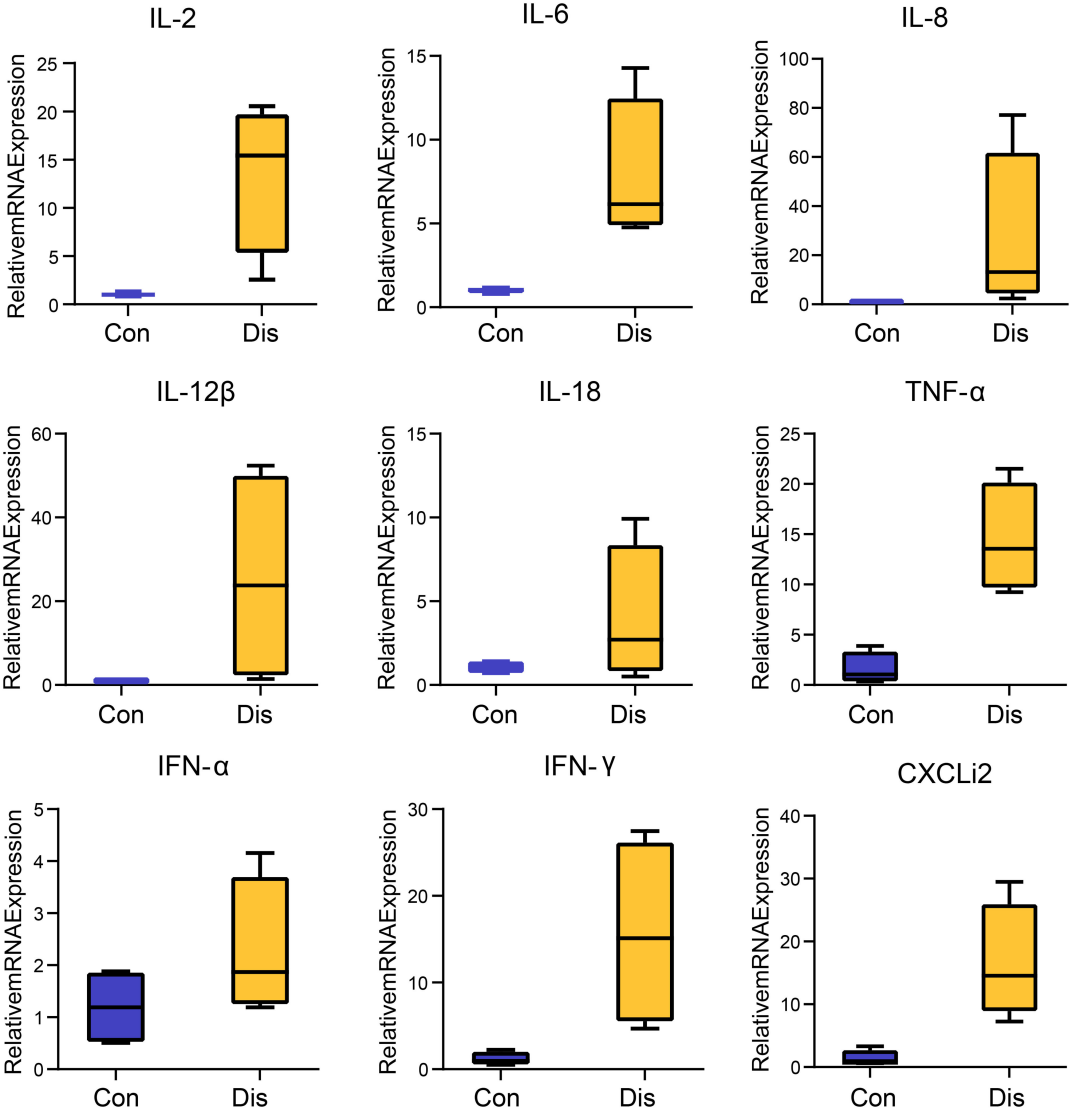
Box–whisker plots of the cytokines expression in the chicken's bursa of Fabricius. Each value indicates the mean ± SEM, *n* = 4.

Given that the immune organ is usually associated with variations in the expression levels of cytokines in viral infection states, we aimed to test whether NIBV infection promoted BF proinflammatory expression. Cytokines-related gene expression profiling of the BF of chickens infected with NIBV was evaluated. As shown in Figure 1, the mRNA expression of inflammatory cytokines, including interleukin (IL)-2 (*P* < 0.05), IL-6 (*P* < 0.05), tumor necrosis factor (TNF)-α (*P* < 0.01), interferon (IFN)-γ (*P* < 0.05) and CXCLi2 (*P* < 0.01), in the chicken's BF was significantly increased in the Dis group in comparison with the Con group.

### Metabolome Profile of the Chicken's BF With NIBV Infection

**Figure 2:**
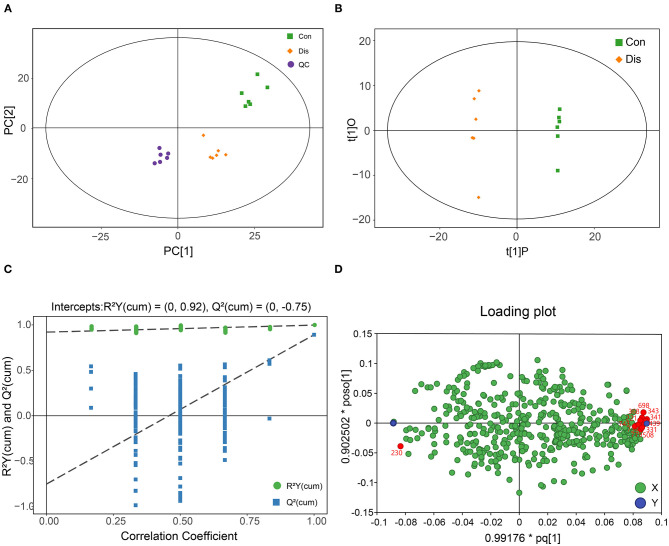
Metabolic profile of chicken infected with NIBV obtained through GC-TOF/MS-based metabolomics. **(A)** The plot of the PCA model. **(B)** The scatter plot of the OPLS-DA model. **(C)** The plot of the 200-permutation test of the OPLS-DA model. **(D)** Loading plot of the bursa of Fabricius samples obtained from chickens, and red points indicate the potential biomarkers.

In order to distinguish the different groups, PCA was first performed on the metabolite data. Figure 2A shows the two-dimensional scatter plots of the PCA model of the QC, Con, and Dis groups. The closely positioned six QC samples also illustrate the good compatibility of the PCA model. In addition, PCA showed that the Con group and the Dis group were completely separated, indicating that NIBV infection significantly changed the metabolite profile. The data are further supervised by OPLS-DA. This model filters the orthogonal signals related to the Y variable (infection variable), so that it can get a highly corrected Y variable, thereby obtaining a better model. As shown in Figure 2B, the OPLS-DA model can perfectly distinguish the two groups, revealing that the metabolite profile of the BF was significantly altered with NIBV infection. At the same time, the data results showed that in the OPLS-DA model of the Con and Dis groups, the values of R2Y = 0.998, R2X = 0.346, and Q2 = 0.892 were obtained. The 200 permutation test was further applied to validate the OPLS-DA model, and the result was shown in Figure 2C. The Q2 intercept value in the model was −0.75, and the low values of the Q2 intercept indicate the robustness of the models and a low risk of over fitting. In addition, a loading plot was constructed based on the OPLS-DA model, which showed the contribution of variables to differences (Figure 2D). Each point in the loading plot represents a metabolite, and the dot far from the origin was considered to have a higher contribution to the model classification. The X variables located near the dummy Y variables have the highest discriminatory power between the categories.

### Differential Metabolites in Response to NIBV Infection

**Figure 3:**
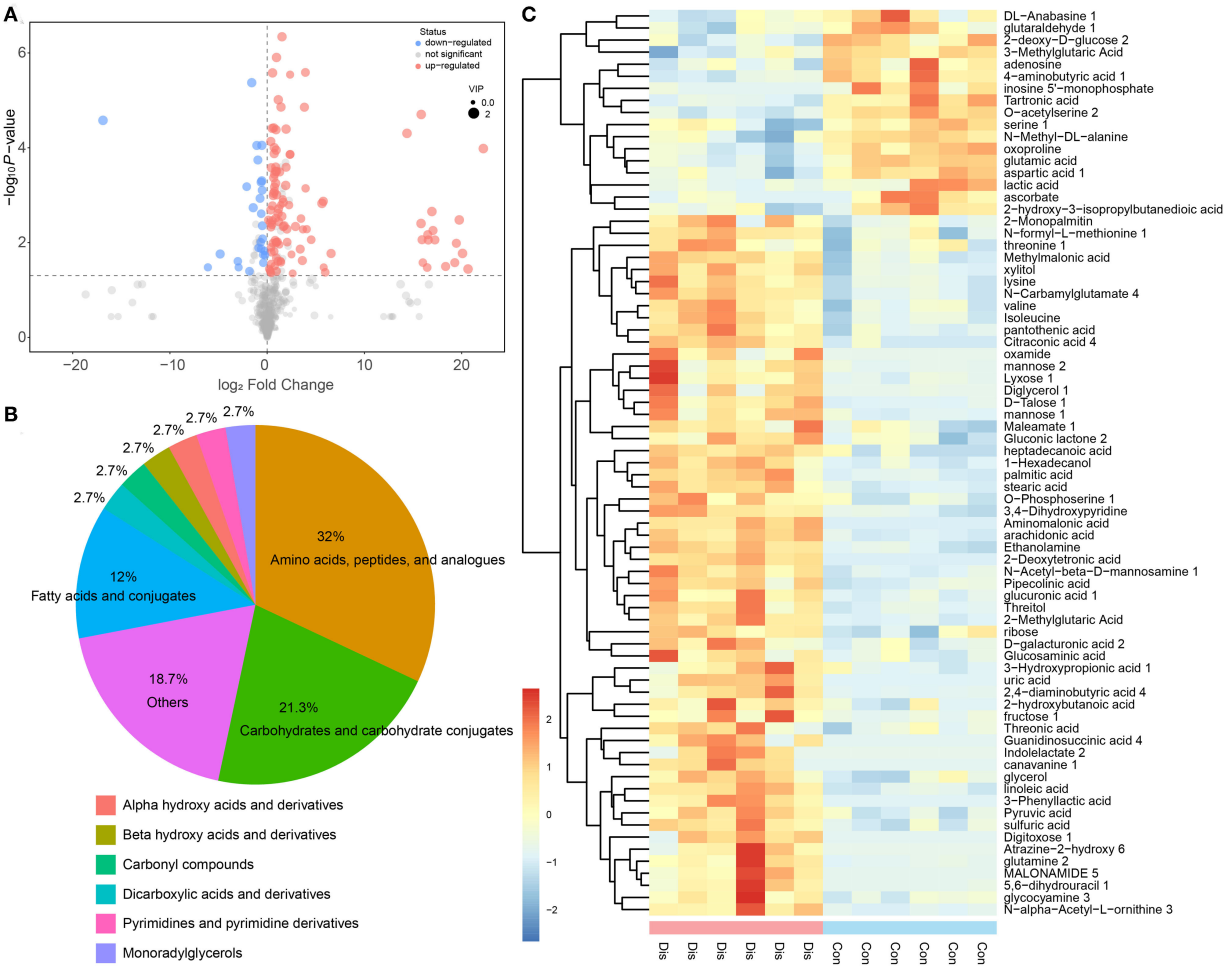
The profile of discrepant metabolites obtained from the OPLS-DA model of the Dis vs. Con group by the selected rule (VIP >1 and *P* < 0.05). **(A)** Volcano plot of differential metabolites in the Dis group compared with the Con group; each point in the volcanic plot represents a metabolite and the size of the point showing the value of the VIP. **(B)** The category of the differential metabolites. **(C)** The heat map representation of unsupervised hierarchical clustering applied in differential metabolites.

The difference in metabolites between the Dis group and the Con group is the most important factor in revealing the coping mechanism of chickens infected with NIBV or the occurrence of damage. To complete this analysis, the first principal component of variable importance projection (VIP) was obtained. Then, in this study, according to the Student's *T*-test, the different metabolites were screened with VIP >1 and *P* < 0.05 as standards. The results of discrepant metabolites in the Dis group compared with the Con group were visualized in the form of volcano plots (Figure 3A). There were 75 annotated differential metabolites in response to NIBV infection, and its categories are shown in Figure 3B. Compared with the Con group, 58 metabolites in the Dis group were upregulated, whereas 17 were downregulated. The annotated differential metabolite profiles in response to NIBV infection were displayed in heat maps (Figure 3C). The number of the upregulated changed metabolites because of the NIBV infection far overstepped the downregulated metabolites.

### Identification of Metabolite Biomarkers

**Figure 4:**
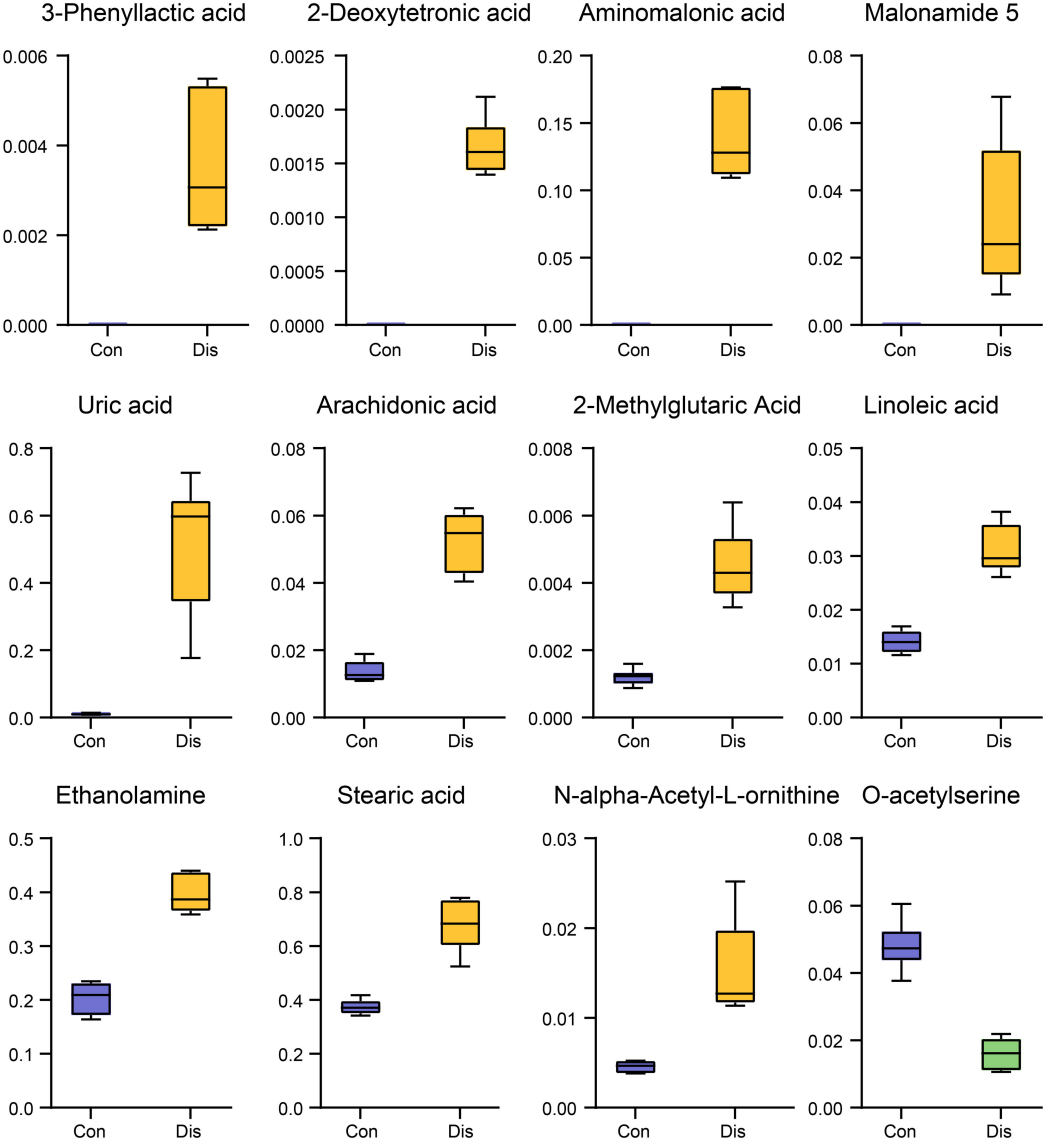
Box–whisker plots of the 12 potential biomarkers in the Dis and Con groups.

To select candidate biomarkers among the differential metabolites, only those metabolites with a high VIP value were considered. The remaining variables were subsequently assessed by Student's *T*-test (*P* < 0.05). Based on these analyses, 12 significantly altered BF metabolites between the groups were obtained, including 3-phenyllactic acid, 2-deoxytetronic acid, aminomalonic acid, malonamide 5, uric acid, arachidonic acid, 2-methylglutaric acid, linoleic acid, ethanolamine, stearic acid, N-alpha-acetyl-l-ornithine, and O-acetylserine. Those potential biomarkers were also shown with red dots in the loading plot of Figure 2D. Furthermore, the box–whisker plots were drawn to improve the visualization changes in the potential biomarkers and describe the distribution of several sets of quantitative data. As presented in Figure 4, the 12 potential biomarkers exhibited differences between the Con and model groups.

### Correlation Study of Inflammatory Cytokines and Metabolite Biomarkers

**Figure 5:**
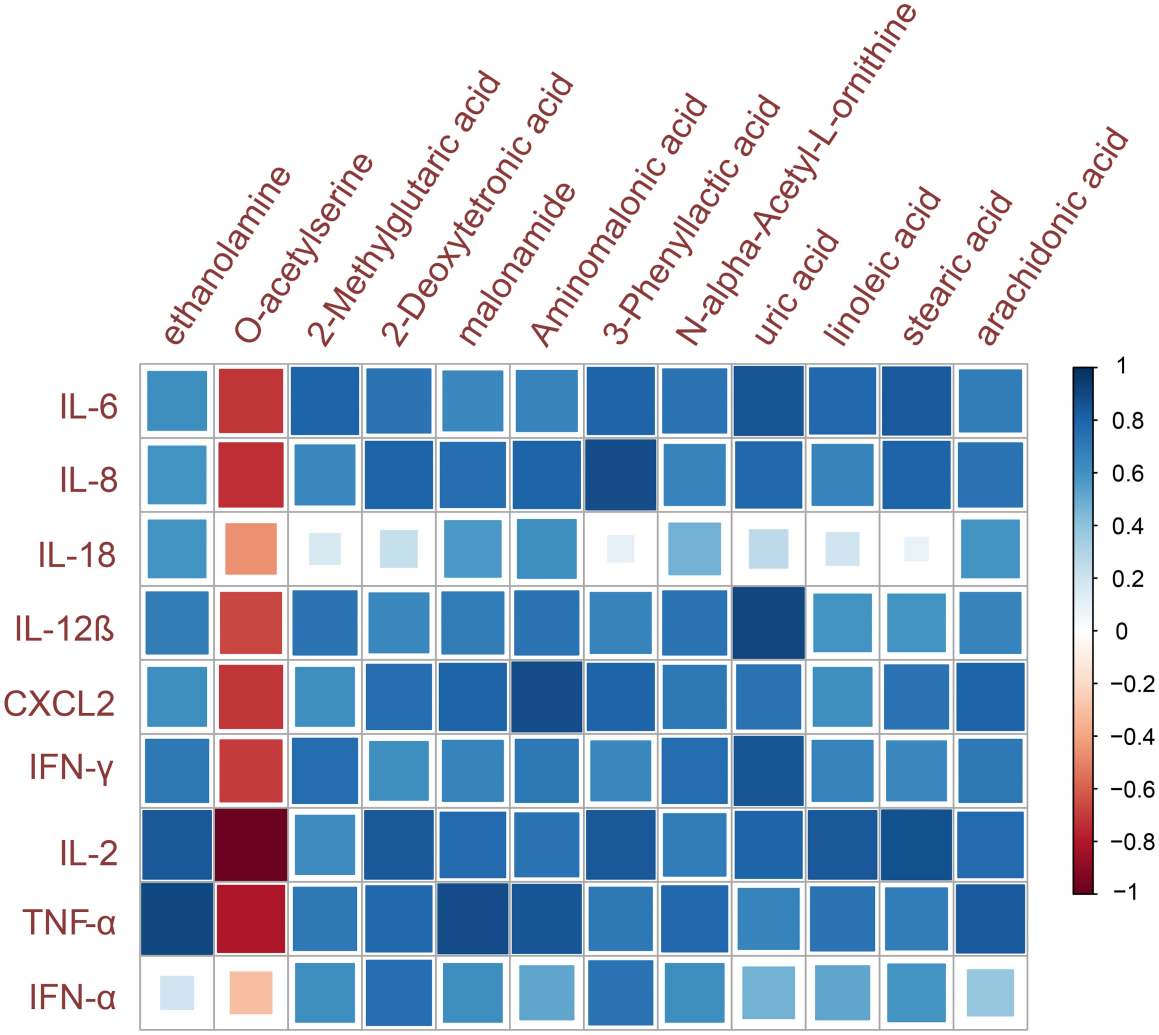
Metabolites and inflammatory cytokines correlation analysis. The color indicates the sign of the correlation, and the size of the square indicates the strength of the correlation (larger square = higher correlation).

The organism's antiviral immune response is related to the extent of the cytokine expression in the case of virus infection. Combined with the above research findings, it is found that virus infection can significantly alter the metabolite profile. Therefore, the relationship between metabolic changes and pathological phenotypes was investigated by evaluating the correlation between the inflammatory cytokines and the abundance of metabolite biomarkers (Figure 5). The results of this cytokine–metabolite correlation study showed that 12 metabolite biomarkers were highly correlated with the expression of at least one inflammatory cytokine mRNA (Spearman correlation coefficient |r| > 0.6). The results showed that most of the selected metabolite biomarkers have a strong positive correlation with inflammatory cytokines, which were significantly increased by IBV infection.

## Discussion

Similar to mammals, avian cytokines play an important role in the host's immune response to pathogen infection (32). Studies by several scholars have shown that severe pathological damages caused by virulent IBV strains in immune organs are related to high levels of virus replication and strong inflammation (33, 34). In the present study, BF is not only an important immune organ of chicken but also a target organ for IBV virus replication. Therefore, systematic investigation of the expression of inflammatory cytokines during NIBV infection can better understand the host immune response to pathogenic infection. Previous studies have found that the biological role of IL-2 is to participate in the growth, activation, and function of immune effector cells, including T cells. The expression level of IL-2 in the body is significantly increased during viral infection, which can rapidly and extremely drive the inflammation of the BF. In addition, IL-6 can cooperate with IL-1 to stimulate T cells to promote the proliferation and differentiation of B cells and the regeneration of platelets (35, 36). We found that NIBV infection caused an increase of IL-2 and IL-6 expression in the chicken's BF. We also found that NIBV infection caused an increase of IL-2 and IL-1β expression in the chicken's BF. Studies have found that viral infection can cause immune organ damage by promoting the expression of IL-2, which is similar to our results (37). IFN-γ is mainly produced by T helper type 1 (Th1) cells, including CD4 T cells, CD8 T cells, and natural killer (NK) cells. It can directly induce and participate in the synthesis of enzymes in the respiratory burst and can enhance the immune response. Similarly, a higher expression level of IFN-γ in the BF was observed in this experiment. Our results are consistent with those reported by De Silva Senapathi et al. who showed that IFN-γ mRNA was significantly increased in the respiratory tract of the chickens with IBV infection (38). Besides the above results, we also observed the upregulation of IL-8, IL-12β, IL-18, TNF-α, IFN-α, and CXCL2 in the BF of NIBV-infected chickens at 10-day post-infection (Figure 1). In mammals, the cytokine storm characterized by dysregulation and exaggeration of inflammatory cytokine production is closely related to the increase in morbidity and mortality of organisms during pathogen infection (39–41). In the present study, NIBV caused vaccinated chickens to reach a peak of death at 10-day post-infection. Based on the above studies, it is shown that the excessive production of cytokines (cytokine storm) caused by NIBV infection plays an important role in the effective elimination of the viruses in chickens. However, at the same time, this is likely to cause serious immune pathological damage to the organism and even cause death.

In our research, based on but not limited to the study of single metabolites, the metabolites were analyzed in multivariate statistical analysis, such as PCA and OPLS-DA, to understand the changes in the BF metabolism of chickens with NIBV infection. The result of OPLS-DA and hierarchical clustering showed that the metabolite profiles between the Con group and the Dis group were significantly different, and that combined with the classification of metabolites, it can be found that the differential metabolites can be mainly classified into amino acids, carbohydrates, and fatty acids (Figure 3). As we all know, amino acids are the most important for organisms, because they can not only be used in the synthesis of proteins and other bioactive molecules but also provide the raw material for most of the synthesis of the cytokine. Nowadays, many metabolomic studies have found that virus infection can upregulate the synthesis of amino acids in organisms (42, 43). Similar results were obtained in this experiment: NIBV infection increased the production of valine, isoleucine, lysine, methionine, glutamine, and threonine. The increase of the amino acid content in the organism amino acid pools contributes to the synthesis of viral proteins and cytokines. In other words, the alteration of metabolite profiling by NIBV infection in the chicken's BF provides ideas for exploring how virus infection can promote self-proliferation by regulating host metabolism.

Metabolomics can fill a key gap in the study of disease systems biology by detecting changes in the abundance of metabolites in organisms related to diseases or disturbances by external factors. In the past, metabolomic studies related to animal models of viral infections focused on the discovery of biomarkers, often neglecting the important information about infection and outcome provided by the metabolite profile (44, 45). In our research, we provided 12 potential biomarkers for distinguishing NIBV infection (Figure 4). These biomarkers mapped to four metabolic pathways, including biosynthesis of unsaturated fatty acids, biosynthesis of amino acids, glycerophospholipid metabolism, and purine metabolism. The synthesis of fatty acids is required for viral infection and/or the maturation of infectious viruses because they are structural elements of cell membranes and viral membranes (46). Viruses target lipid synthesis and signal transduction to remodel their host cells and generate lipids for the virus envelope (47). In this study, the biomarkers mapped to the biosynthesis of unsaturated fatty acids are arachidonic acid, linoleic acid, and stearic acid. Interestingly, there are many other reports using arachidonic acid as a biomarker of viral infection, such as hepatitis B virus, hepatitis C virus, and influenza A (H1N1) (48–50). Previous studies have shown that the virus regulates purine and nucleotide synthesis and metabolism, including the *de novo* synthesis pathways or salvage synthesis pathways for its own nucleic acid synthesis, which leads to an increase in uric acid in the purine metabolism pathway (43, 51). Uric acid may be an important biomarker for NIBV infection because it causes mitochondrial dysfunction and endoplasmic reticulum stress, which lead to increased lipogenesis and overexpression of lipase, respectively (52, 53). Furthermore, metabolomic analysis can help understand the pathogenesis of diseases from the perspective of the metabolic pathways and can be used to discover the potential of new biomarkers, whereas other high-throughput methods may ignore these molecules. Therefore, metabolomics also has the potential to discover new, unexpected metabolic pathways to participate in viral infections, pathogenesis, and physiological responses to anti-viral strategies.

Interestingly, the results of the correlation analysis between biomarkers and inflammatory cytokines in this experiment show that there is a strong correlation (Figure 5). Similarly, recent research pointed out that the metabolic pathways have recently been generally involved in directing the immune response, which may play an important role in the pathogenesis of viral infection-related diseases. For example, it was shown that the tricarboxylic acid (TCA) cycle intermediates directly bind to dendritic cells, causing the production of proinflammatory cytokines, and the level of intracellular succinate has an important regulatory effect on the production of IL-1β in macrophages activated by Toll-like receptor agonists (54). Based on the above findings, the role of metabolic pathways in immune function may become another research hotspot in the field of biology.

## Data Availability Statement

The original contributions generated in the study are included in the article/Supplementary Materials, further inquiries can be directed to the corresponding authors.

## Ethics Statement

The animal study was reviewed and approved by Institutional Animal Care and Use Committee of Jiangxi Agricultural University (Approval ID: JXAULL-2017003).

## Author Contributions

XG: conceptualization, project administration, and funding acquisition. XG and PL: methodology. CZ and YS: software. SC and YY: validation. PX, CZ, and JK: formal analysis. GL, PL, and XG: resources. PX and XG: data curation. PX and JK: writing—original draft preparation. XG, JK, PX, YZ, RH, GL, and GH: writing—review and editing. PX, CZ, and YS: visualization. GH: supervision. All authors have read and agreed to the published version of the manuscript.

## Conflict of Interest

The authors declare that the research was conducted in the absence of any commercial or financial relationships that could be construed as a potential conflict of interest.
