# Supplementary material for: Nephropathogenic Infectious Bronchitis Virus Infection Altered the Metabolome Profile and Immune Function of the Bursa of Fabricius in Chicken
Source: Front Vet Sci. 2021 Jan 21;7:628270. doi: 10.3389/fvets.2020.628270 (PMC7858655; doi:10.3389/fvets.2020.628270)
Supplement: Supplementary file 1 [file Table_1.DOCX]

**Supplementary Table. Differentially abundant metabolites identified by GC-TOF/MS. Significant differences were declared at the level of *P*-value < 0.05 and VIP > 1.**

| **Peak** | **Similarity** | **rt** | **Count** | **Mass** | **MEAN Dis** | **MEAN**  **Con** | **VIP** | **P-VALUE** | **Q-VALUE** | **FOLD CHANGE** | **LOG FOLDCHANGE** |
| --- | --- | --- | --- | --- | --- | --- | --- | --- | --- | --- | --- |
| threonine 1 | 946 | 12.0667,0 | 30 | 219 | 0.889687163 | 0.722799885 | 1.191965308 | 0.038622084 | 0.045510069 | 1.230890017 | 0.299701859 |
| valine | 944 | 9.73336,0 | 30 | 144 | 2.096885471 | 1.437867774 | 1.610834801 | 0.001670749 | 0.004964287 | 1.458329833 | 0.544317054 |
| Isoleucine | 940 | 10.8386,0 | 30 | 158 | 1.266462016 | 0.746726167 | 1.751425092 | 0.00036055 | 0.001805908 | 1.696019335 | 0.762152617 |
| palmitic acid | 935 | 19.5164,0 | 30 | 117 | 0.733785354 | 0.462229458 | 1.867874805 | 0.000905053 | 0.003324709 | 1.587491541 | 0.666748904 |
| serine 1 | 933 | 11.7403,0 | 30 | 73 | 4.240840179 | 5.049703862 | 1.372500271 | 0.014994149 | 0.022713582 | 0.839819581 | -0.25184867 |
| D-Talose 1 | 927 | 17.8572,0 | 30 | 319 | 0.043929285 | 0.015470193 | 1.878635404 | 0.002788474 | 0.007242682 | 2.839608137 | 1.505691853 |
| stearic acid | 926 | 21.3162,0 | 30 | 117 | 0.677703553 | 0.373912188 | 1.914855158 | 0.000313779 | 0.001639782 | 1.812467137 | 0.857954837 |
| glutamic acid | 925 | 14.9919,0 | 30 | 246 | 0.46536646 | 0.973856421 | 1.756278736 | 8.92771E-05 | 0.000782401 | 0.477859416 | -1.065341849 |
| aspartic acid 1 | 919 | 13.8268,0 | 30 | 232 | 1.194606125 | 1.749764161 | 1.673824773 | 0.000496162 | 0.002264254 | 0.682724079 | -0.550625461 |
| Pyruvic acid | 918 | 7.33097,0 | 30 | 174 | 0.032199754 | 0.020149669 | 1.760737761 | 0.000429657 | 0.002050125 | 1.598028899 | 0.676293498 |
| N-Methyl-DL-alanine | 917 | 9.10086,0 | 30 | 130 | 0.036835148 | 0.052185676 | 1.57614712 | 0.004411976 | 0.009708494 | 0.705847872 | -0.502570815 |
| Ethanolamine | 913 | 10.4532,0 | 30 | 174 | 0.395888949 | 0.203509739 | 1.916574127 | 1.24388E-06 | 9.43157E-05 | 1.945307146 | 0.959997962 |
| 2-hydroxybutanoic acid | 912 | 8.42903,0 | 30 | 131 | 0.031858565 | 0.016698967 | 1.551520159 | 0.010077373 | 0.016986321 | 1.907816468 | 0.931922391 |
| ribose | 893 | 15.6039,0 | 30 | 103 | 0.081523562 | 0.062019891 | 1.489792688 | 0.004723107 | 0.010097451 | 1.314474447 | 0.394486097 |
| lysine | 893 | 18.2771,0 | 30 | 174 | 0.58008985 | 0.238409819 | 1.742595239 | 0.000323084 | 0.001665846 | 2.433162577 | 1.28283272 |
| linoleic acid | 884 | 21.0362,0 | 29 | 337 | 0.031148771 | 0.014090785 | 1.924701104 | 9.69207E-06 | 0.000220928 | 2.210577422 | 1.144423263 |
| mannose 2 | 883 | 18.1856,0 | 30 | 160 | 0.183867062 | 0.014965925 | 1.751838825 | 0.023927243 | 0.031846459 | 12.28571313 | 3.618909697 |
| mannose 1 | 880 | 17.9875,0 | 30 | 157 | 0.269102579 | 0.035751267 | 1.760651613 | 0.004573111 | 0.009912735 | 7.527078073 | 2.912089936 |
| glycerol | 878 | 10.5739,0 | 30 | 205 | 0.253423675 | 0.197820494 | 1.617904033 | 0.002057035 | 0.005816313 | 1.281078967 | 0.357359408 |
| pantothenic acid | 873 | 18.9455,0 | 30 | 291 | 0.022903472 | 0.011731507 | 1.749139697 | 0.000313323 | 0.001638489 | 1.952304248 | 0.9651779 |
| lactic acid | 857 | 7.49122,0 | 30 | 59 | 0.237884317 | 0.840147129 | 1.462765568 | 0.040170789 | 0.046811297 | 0.283146022 | -1.820381836 |
| xylitol | 855 | 16.0839,0 | 30 | 217 | 0.034718043 | 0.015502612 | 1.780044863 | 0.001949641 | 0.005587824 | 2.239496402 | 1.163174348 |
| Threitol | 845 | 13.6103,0 | 30 | 217 | 0.028762011 | 0.013247422 | 1.809743352 | 0.0024025 | 0.006519855 | 2.171140239 | 1.118452916 |
| fructose 1 | 843 | 17.7018,0 | 30 | 103 | 0.014273121 | 0.00736477 | 1.547410671 | 0.040475526 | 0.047063955 | 1.938026618 | 0.954588385 |
| Aminomalonic acid | 839 | 13.1597,0 | 23 | 218 | 0.138447478 | 2.80703E-08 | 2.028470056 | 0.000103834 | 0.000847726 | 4932171.321 | 22.23379148 |
| arachidonic acid | 834 | 22.3783,0 | 29 | 80 | 0.052554725 | 0.013643445 | 1.962777133 | 4.03001E-05 | 0.000480499 | 3.852012722 | 1.945612468 |
| oxoproline | 824 | 13.8995,0 | 30 | 258 | 0.918947787 | 1.341985152 | 1.827465681 | 8.88447E-05 | 0.000780314 | 0.684767477 | -0.546313912 |
| N-Acetyl-beta-D-mannosamine 1 | 820 | 19.7701,0 | 30 | 202 | 0.020414114 | 0.011141072 | 1.869175468 | 0.000561137 | 0.002456168 | 1.83232951 | 0.873678968 |
| uric acid | 820 | 19.9851,0 | 30 | 442 | 0.519281635 | 0.010420663 | 2.001617419 | 0.001488365 | 0.00461974 | 49.83191894 | 5.638998226 |
| inosine 5'-monophosphate | 817 | 26.9746,0 | 23 | 169 | 0.000780627 | 0.022275168 | 1.784821768 | 0.017404315 | 0.025243455 | 0.035044729 | -4.834658704 |
| adenosine | 801 | 24.4401,0 | 30 | 236 | 0.0052664 | 0.007267651 | 1.463749233 | 0.008297195 | 0.014978294 | 0.724635818 | -0.464671976 |
| Threonic acid | 772 | 14.035,0 | 30 | 292 | 0.007320489 | 0.005423488 | 1.360414158 | 0.016019746 | 0.023817075 | 1.349775035 | 0.432718975 |
| O-Phosphoserine 1 | 754 | 17.3578,0 | 27 | 356 | 0.003227117 | 0.000678255 | 1.292321686 | 0.001618829 | 0.004869238 | 4.757969717 | 2.25034609 |
| 4-aminobutyric acid 1 | 732 | 13.9396,0 | 30 | 304 | 0.002432583 | 0.003730999 | 1.765036168 | 0.000531211 | 0.002369763 | 0.651992464 | -0.617072806 |
| 2-Monopalmitin | 732 | 23.8267,0 | 27 | 218 | 0.008135067 | 0.004918979 | 1.371022648 | 0.015320221 | 0.023068915 | 1.653812066 | 0.7257953 |
| D-galacturonic acid 2 | 663 | 18.651,0 | 30 | 292 | 0.005195702 | 0.002720271 | 1.487786985 | 0.009773121 | 0.0166622 | 1.909994286 | 0.933568322 |
| heptadecanoic acid | 648 | 20.4265,0 | 26 | 117 | 0.0068316 | 0.001319746 | 1.48046017 | 0.00013698 | 0.000967619 | 5.17644887 | 2.371962725 |
| oxamide | 629 | 11.3427,0 | 11 | 102 | 0.018072959 | 2.80703E-08 | 1.692915664 | 0.026524059 | 0.034271447 | 643846.531 | 19.29635732 |
| 2-Deoxytetronic acid | 629 | 12.6588,0 | 17 | 231 | 0.001652925 | 2.80703E-08 | 2.028781117 | 1.98025E-05 | 0.000337984 | 58885.21079 | 15.84561772 |
| ascorbate | 628 | 18.4935,0 | 12 | 332 | 0.000780222 | 0.005976889 | 1.124677098 | 0.033375053 | 0.040876557 | 0.130539845 | -2.937437864 |
| 3-Phenyllactic acid | 602 | 14.5772,0 | 18 | 193 | 0.003538671 | 2.80703E-08 | 2.029537777 | 0.002212109 | 0.006139282 | 126064.6286 | 16.94380401 |
| sulfuric acid | 580 | 9.04314,0 | 30 | 216 | 0.06913409 | 0.051498618 | 1.789069671 | 0.000263283 | 0.001485067 | 1.342445525 | 0.424863547 |
| Indolelactate 2 | 570 | 20.6624,0 | 19 | 202 | 0.002524966 | 2.80703E-08 | 1.447545172 | 0.033602438 | 0.041084909 | 89951.53833 | 16.45686033 |
| Methylmalonic acid | 560 | 9.46156,0 | 30 | 147 | 0.225080106 | 0.173635053 | 1.78862063 | 7.34631E-05 | 0.000699621 | 1.296282643 | 0.374380319 |
| 1-Hexadecanol | 528 | 18.6772,0 | 28 | 299 | 0.00391379 | 0.002067458 | 1.839370603 | 0.000111921 | 0.000880294 | 1.893044027 | 0.920707965 |
| Pipecolinic acid | 517 | 11.8135,0 | 30 | 156 | 0.014088557 | 0.003651249 | 1.705763011 | 0.000257232 | 0.001464865 | 3.858558765 | 1.948062078 |
| MALONAMIDE 5 | 496 | 12.6889,0 | 24 | 89 | 0.031403883 | 2.80703E-08 | 2.02678506 | 0.016909767 | 0.024742044 | 1118758.767 | 20.09346756 |
| Tartronic acid | 495 | 11.8616,0 | 30 | 102 | 0.008094422 | 0.021903921 | 1.823106873 | 0.001835497 | 0.005338042 | 0.369542162 | -1.43618912 |
| DL-Anabasine 1 | 477 | 12.1599,0 | 30 | 70 | 0.013921246 | 0.018354534 | 1.297711594 | 0.026735866 | 0.034463004 | 0.758463618 | -0.398848116 |
| Glucosaminic acid | 475 | 19.2674,0 | 30 | 318 | 0.059579647 | 0.03959803 | 1.478702242 | 0.010262713 | 0.017180191 | 1.504611411 | 0.589390937 |
| Lyxose 1 | 457 | 15.3031,0 | 29 | 204 | 0.00360754 | 0.000636062 | 1.02966484 | 0.0177822 | 0.025620726 | 5.671677983 | 2.503775624 |
| N-Carbamylglutamate 4 | 453 | 19.3447,0 | 29 | 174 | 0.027976396 | 0.010448054 | 1.852239025 | 1.38065E-05 | 0.000270013 | 2.677665804 | 1.420975911 |
| 2-deoxy-D-glucose 2 | 446 | 16.7263,0 | 30 | 319 | 0.002522535 | 0.004191673 | 1.647969599 | 0.001166858 | 0.003931084 | 0.601796782 | -0.732651702 |
| 3-Methylglutaric Acid | 445 | 12.5608,0 | 30 | 172 | 0.014543332 | 0.020742107 | 1.579124108 | 0.000786489 | 0.003039413 | 0.70115016 | -0.512204646 |
| 2-hydroxy-3-isopropylbutanedioic acid | 441 | 14.4459,0 | 30 | 247 | 0.005686615 | 0.008694663 | 1.36762644 | 0.013488451 | 0.021025727 | 0.654035159 | -0.612559901 |
| glycocyamine 3 | 436 | 17.5343,0 | 30 | 217 | 0.002865517 | 0.001079791 | 1.503379477 | 0.025971003 | 0.033766934 | 2.65377036 | 1.408043535 |
| 3-Hydroxypropionic acid 1 | 422 | 8.7495,0 | 30 | 234 | 0.004968151 | 0.002803465 | 1.569819065 | 0.008950139 | 0.015747071 | 1.772146603 | 0.825497958 |
| 5,6-dihydrouracil 1 | 422 | 13.3284,0 | 25 | 100 | 0.044312227 | 0.000465499 | 1.819272469 | 0.016965694 | 0.024799188 | 95.19295226 | 6.57278286 |
| Maleamate 1 | 406 | 14.333,0 | 30 | 244 | 0.004476515 | 0.003379717 | 1.387285783 | 0.012574912 | 0.019985892 | 1.324523527 | 0.40547347 |
| Diglycerol 1 | 370 | 16.4201,0 | 24 | 205 | 0.001903461 | 3.3851E-05 | 1.06732094 | 0.033359962 | 0.040862704 | 56.23065273 | 5.813284889 |
| O-acetylserine 2 | 364 | 10.6461,0 | 30 | 102 | 0.015995221 | 0.048058253 | 1.892748243 | 4.22877E-06 | 0.000123555 | 0.332829854 | -1.58714325 |
| 3,4-Dihydroxypyridine | 359 | 13.0835,0 | 30 | 256 | 0.003255554 | 0.002025392 | 1.847940005 | 3.8046E-05 | 0.000469175 | 1.607370366 | 0.684702389 |
| 2,4-diaminobutyric acid 4 | 333 | 14.9653,0 | 29 | 331 | 0.048594136 | 0.002893103 | 1.202963333 | 0.005214459 | 0.010788013 | 16.79654456 | 4.070092562 |
| canavanine 1 | 323 | 19.0176,0 | 14 | 204 | 0.004384149 | 2.80703E-08 | 1.710216038 | 0.008778722 | 0.015549046 | 156184.6764 | 17.25289339 |
| Citraconic acid 4 | 320 | 11.5979,0 | 23 | 292 | 0.000793132 | 5.57317E-05 | 1.753063013 | 1.39232E-05 | 0.000271202 | 14.2312421 | 3.83098968 |
| Gluconic lactone 2 | 298 | 18.078,0 | 24 | 208 | 0.015276117 | 0.008269625 | 1.494928141 | 0.003025507 | 0.00765672 | 1.847256373 | 0.885384106 |
| glutamine 2 | 292 | 16.0949,0 | 30 | 216 | 0.00362149 | 0.000343347 | 1.699451934 | 0.01371546 | 0.021277947 | 10.54760414 | 3.398843426 |
| N-alpha-Acetyl-L-ornithine 3 | 290 | 15.8142,0 | 29 | 188 | 0.015287808 | 0.004572958 | 1.906486113 | 0.004430985 | 0.00973291 | 3.343089469 | 1.741181965 |
| Atrazine-2-hydroxy 6 | 287 | 18.5926,0 | 16 | 357 | 0.009408152 | 2.80703E-08 | 1.72958467 | 0.031889947 | 0.039498065 | 335164.0435 | 18.35450786 |
| N-formyl-L-methionine 1 | 262 | 16.0023,0 | 22 | 272 | 0.002843986 | 0.001075243 | 1.012511124 | 0.00160898 | 0.004850942 | 2.644969775 | 1.403251236 |
| Guanidinosuccinic acid 4 | 261 | 19.9537,0 | 23 | 398 | 0.020185887 | 2.80703E-08 | 1.70347013 | 0.010334886 | 0.017254968 | 719119.3022 | 19.45587161 |
| glucuronic acid 1 | 259 | 17.9091,0 | 29 | 304 | 0.003718962 | 0.001182067 | 1.816585375 | 0.004596686 | 0.009942107 | 3.146152031 | 1.653588388 |
| Digitoxose 1 | 246 | 14.9958,0 | 17 | 69 | 0.003827823 | 2.80703E-08 | 1.729034523 | 0.005558486 | 0.011278318 | 136365.6286 | 17.05712053 |
| glutaraldehyde 1 | 240 | 8.87417,0 | 30 | 174 | 0.024858458 | 0.033000708 | 1.357515632 | 0.018077841 | 0.02591242 | 0.753270443 | -0.408760172 |
| 2-Methylglutaric Acid | 215 | 12.4972,0 | 28 | 172 | 0.004505733 | 0.00120459 | 1.950669089 | 0.000622198 | 0.002622757 | 3.740469404 | 1.90321933 |
